# Infection control approaches to group a *Streptococcus* outbreaks in behavioral health settings

**DOI:** 10.1017/ash.2025.10106

**Published:** 2025-08-15

**Authors:** Kevin M. Gibas, Briana Castro, Kavyasri Melapu, Maria F. Gomes, Michael P. Koster

**Affiliations:** 1 Department of Epidemiology & Infection Prevention, Rhode Island Hospital, Providence, RI, USA; 2 Warren Alpert Medical School of Brown University, Providence, RI, USA; 3 Department of Epidemiology & Infection Prevention, Mass General Brigham, Boston, MA, USA; 4 College of Public Health, Kent State University, Kent, OH, USA; 5 Department of Epidemiology & Infection Prevention, Emma Pendleton Bradley Hospital, Providence, RI, USA

## Introduction

The COVID-19 pandemic significantly increased awareness of infection prevention and control (IPC) in behavioral health settings.^1^ Beyond respiratory infections, such as with SARS-CoV-2, there are other IPC challenges in behavioral health settings, including risk of infections and outbreaks with organisms such as Group A Streptococcus (GAS).^2–4^ Factors such as the milieu treatment environment, communal dining, hands-on care, poor symptom reporting, patients’ inability to follow certain protocols, and ligature risks associated with interventions create barriers to effective infection containment.

In 2023, invasive GAS infections rates reached a 20-year high in the United States.^5^ During this time, our IPC team began to observe an increase in GAS pharyngitis cases and associated outbreaks in behavioral health units (BHUs). In 2024 our adult BHU experienced its first GAS outbreak in several years, prompting the unit to ask for guidance from the IPC team. While navigating these GAS outbreaks, we discovered our IPC department lacked a standardized approach for managing GAS outbreaks. To address this gap, we constructed epidemic curves for GAS pharyngitis cases in our BHUs for 2024 and conducted a literature review to inform the development of standard workflow and mitigation strategies. Here we present the results of our epidemiologic investigation and literature review as well as mitigation strategies for GAS outbreaks in BHUs.

## Methods

We conducted an epidemiologic investigation of GAS pharyngitis cases in our BHUs during 2024. Confirmed cases were defined as patients with symptoms of pharyngitis or tonsillitis plus positive throat rapid antigen detection test (RADT) or polymerase chain reaction (PCR) tests for GAS; probable cases had similar symptoms without laboratory confirmation. An outbreak was defined as two or more confirmed or probable cases in the same BHU within three days of the index case’s symptom onset. All microbiology tests, including RADTs and PCRs, are integrated into electronic clinical surveillance software which we queried for positive GAS results from BHU patients and plotted chronologically to create an epidemic curve.

Using this data, we aimed to develop standard workflow and mitigation plans for GAS outbreaks. To inform these documents, we conducted a narrative literature review for GAS outbreaks in behavioral health settings. We searched PubMed, Embase, Web of Knowledge, and Google Scholar (July 22–August 6, 2024), using MeSH terms related to *Streptococcus pyogenes*, infection control, and behavioral health settings. The search was limited to English-language articles, and our team selected the 10 most relevant studies from the review.

Based on this review, we developed preliminary standardized workflow and mitigation documents to support the IPC team and frontline staff in responding to GAS pharyngitis outbreaks. These were subsequently reviewed and refined by the IPC team—including hospital epidemiologists and infection preventionists—to finalize the standard workflow and mitigation protocols.

## Results

**Figure 1.**
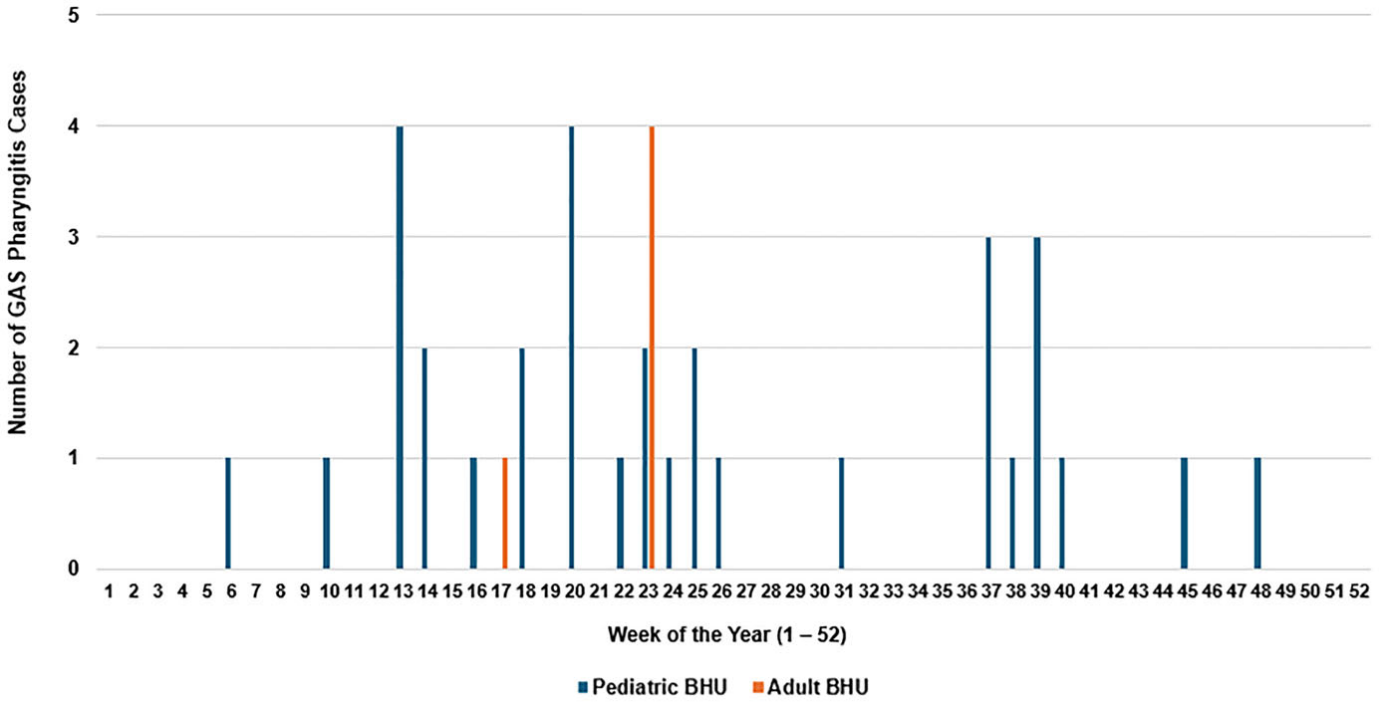
Group a strep pharyngitis cases diagnosed in behavioral health units (BHUs) at Brown University Health in 2024.

In 2024, we identified 38 GAS pharyngitis cases in our BHUs—33 in pediatric patients and 5 in adults (Figure 1). There were 9 distinct GAS outbreaks in 2024: 8 occurred in pediatric/adolescent BHUs and 1 in an adult BHU. All four pediatric/adolescent BHUs and one of five adult BHUs were affected. The average age was 10 years for pediatric cases and 33 years for adults. Most infections occurred in males (61% of pediatric and 80% of adult cases). The mean time from admission to diagnosis was 29 days for pediatric/adolescent patients (range: 2–83 d) and 36 days for adults (range: 2–142 d).

Our literature review revealed a predominant focus on COVID-19, with limited data on managing other infectious outbreaks in these settings. Few studies addressed IPC strategies for GAS, particularly in behavioral health settings.^2,3^ Our review identified limited research addressing GAS in geriatric and long-term care facilities.^6–8^ Common themes we identified included the need for IPC practices focused on early case detection, staff and patient education, active surveillance, and environmental controls that consider the unique needs and constrains of the treatment setting and patient population when managing outbreaks in behavioral health settings.^1–10^

Using insights from our literature review and feedback from our IPC team, we developed standard workflow and mitigation documents for managing GAS outbreaks (Supplemental Figure 1). The workflow document includes key definitions (eg, confirmed and probable GAS cases, outbreak and exposed population definitions), isolation guidance, the infectious period of GAS, and notification protocols for positive cases and potential outbreaks. The GAS exposure standard workflow emphasizes the importance of timely implementation and consistent adherence to contact and droplet isolation precautions for infectious individuals. The mitigation plan provides specific guidance regarding isolation precautions, contact tracing, staffing strategies, employee illness reporting, patient and staff testing, hand hygiene promotion, enhanced cleaning procedures, patient cohort isolation, visitation policies, and ultimately containing the outbreak. Decisions regarding active surveillance (patient screening for GAS carriage), enhanced cleaning procedures, and visitation policies are made in collaboration with the IPC team and BHU staff, based on the specific context of the outbreak.

These documents are shared electronically during outbreaks and serve as practical resources for the IPC team and frontline staff during GAS outbreaks. Since their development, these tools have been adopted as standard practice for managing GAS outbreaks at our organization. They have been utilized in several subsequent outbreaks within our BHUs and have received positive feedback from both IPC and frontline BHU staff.

## Discussion

Behavioral health settings present significant IPC challenges for highly-contagious infections like GAS, underscored by recent national increases in cases and the outbreaks observed in our BHUs.^3,9,10^ Our investigation demonstrated the incidence of GAS pharyngitis in BHUs within a large academic healthcare system and revealed that most affected individuals—particularly pediatric and adolescent patients—had prolonged hospitalizations prior being diagnosed with GAS. Our findings underscore the need for robust, standardized IPC strategies tailored to these unique care environments. The absence of preexisting guidance for managing GAS outbreaks in behavioral health settings previously hindered the ability of our IPC team and frontline hospital staff to quickly and efficiently respond to outbreaks.

The standardized workflow and mitigation resources we developed to address these needs provide clarity on roles, responsibilities, and procedures, ensuring that all team members are aligned in their approach to managing outbreaks. Further, these tools assist IPC and BHU staff with managing outbreaks by establishing outbreak criteria and providing guidance for case identification, contact tracing, visitation policies, staffing adjustments, staff illness reporting, illness reporting, enhanced cleaning, hygiene promotion, and patient cohort isolation. They also provide a clear notification chain to ensure timely communication within the health system and with public health entities. Although developed in response to GAS, the framework is adaptable to other infectious diseases. Successful implementation relies on close collaboration between IPC teams and BHU staff.

Most existing studies on IPC strategies in behavioral health settings focus primarily on respiratory infections, which leaves a significant gap in understanding the management of other infectious diseases in these contexts. Although research from long-term care and geriatric settings offers some insights, further research is essential to develop effective IPC strategies for communicable diseases in behavioral health settings. Despite the unique challenges and limited data on effective approaches for managing outbreaks in behavioral health settings, the use of standardized workflows and mitigation protocols can be useful tools for managing and guiding staff through infectious outbreaks. Future research should emphasize IPC practices specifically tailored to the distinct needs of these treatment settings and their patient population.

## Supporting information

10.1017/ash.2025.10106.sm001 Gibas et al. supplementary material Gibas et al. supplementary material

## Supplementary material

The supplementary material for this article can be found at https://doi.org/10.1017/ash.2025.10106
