# Supplementary material for: Infection control approaches to group a Streptococcus outbreaks in behavioral health settings
Source: Antimicrob Steward Healthc Epidemiol. 2025 Aug 15;5(1):e187. doi: 10.1017/ash.2025.10106 (PMC12394017; doi:10.1017/ash.2025.10106)
Supplement: Gibas et al. supplementary material [file S2732494X2510106Xsup001.pdf]

Supplemental Figure 1: Group A Streptococcal PHARYNGITIS/TONSILITIS Exposure Standard Workflow and Mitigation Plan
Department of Epidemiology and Infection Prevention *

Step/Intervention	Procedure
**Case definition**	• **Confirmed Case:** Lab confirmed Group A Streptococcal (Strep, GAS) pharyngeal infection by either a positive rapid antigen detection test (RADT, rapid strep test) or throat (pharyngeal) culture with Group A Strep in a patient with signs/symptoms consistent with streptococcal pharyngitis/tonsillitis. **<br>• **Probable Case:** Patient with signs, symptoms, and/or exam findings consistent with group A strep pharyngitis in the absence of laboratory confirmation of group A strep infection.
**Infectious period**	• **24 hours prior** to symptom onset until **24 hours after** initiation of appropriate antibiotic therapy.
**Outbreak definition**	• Two or more confirmed or probable cases (based on the above definitions) occurring on the same unit/program within 3 days of onset of signs/symptoms consistent with strep pharyngitis/tonsilitis in the index case.
**Exposed Population Definition**	• Patients sharing the same room, bathrooms, and eating spaces.<br>• Infection Preventionists (IPs)/Infection Prevention Associates (IPAs) should discuss with the Medical Directors about other potentially exposed patients if there is an ongoing outbreak.
**Exposed isolation**	• Asymptomatic exposed patients do not automatically require isolation.<br>• Any symptomatic patient should immediately be placed in contact & droplet isolation and tested for Group A Strep with a rapid strep test (RADT).<br>• Discuss with the Medical Directors if there is a role for universal testing or screening if there is an ongoing outbreak.
**Definition of Immune Population**	• N/A
**Notification**	• Infection Prevention/Control Medical Director(s), Infection Prevention/Control Director/Manager(s), Psychiatrist (BHU) or Attending Physician, Unit Nurse Manager, Risk Management, Department of Health (for ongoing outbreak).
**Mitigation plan**	• See GAS Pharyngitis Cluster Interventions
**Flagging of charts**	• The IPA will flag the patient record: Group A Strep (Strep Pyogenes).<br>• **COMMENT BOX:** Contact & Droplet Isolation until 24hrs after the initiation of treatment.
**Special considerations**	• Educate/remind staff, patients, and visitors of the importance of appropriate hand hygiene practices/compliance.

* This document does not cover management of outbreaks related to group A streptococcal (Strep Pyogenes) infections causing other conditions such as scarlet fever, impetigo, erysipelas, cellulitis, puerperal sepsis, neonatal sepsis, invasive group A strep, streptococcal toxic shock syndrome, or necrotizing fasciitis. For such cases, please consult with the Infection Control Medical Directors.

** Symptoms of Group A strep pharyngitis/tonsillitis include sudden onset of fever, throat pain, difficulty swallowing, exudative tonsillitis, and tender, enlarged anterior cervical lymph nodes. Exam findings consistent with group A strep pharyngitis/tonsillitis include injection/erythema (redness) and edema (swelling) of the tonsils and soft palate with exudate (pus). Patients may also have petechiae (tiny spots of bleeding under the skin) on the soft palate.

Supplemental Figure 1: Group A Streptococcal PHARYNGITIS/TONSILITIS Exposure Standard Workflow and Mitigation Plan
Department of Epidemiology and Infection Prevention *

Group A Strep (GAS) Pharyngitis Cluster Interventions

SITUATION:

BACKGROUND:

ASSESSMENT: Patient/Staff transmission is suspected. A mitigation plan is needed. The Infection Preventionist contact is. Call the operator on off hours.

Mitigation Plan Start Date: **Cluster Resolved:**

RECOMMENDATIONS:

	**Outbreak Mitigation Strategies** *(Select all that Apply)*	**Special Considerations**
	1. **Visitation**	
	a. Unit to remain open to visitation	Visitors must perform hand hygiene before entering the unit and follow isolation sign instructions.
	b. Close unit to visitation	
	2. **Isolation Precautions**	
	a. Patients with suspected or confirmed GAS must be immediately placed on **contact & droplet isolation** in a private room until 24 hours after the start of treatment.	If no private bathroom, provide an alternate bathroom for the connecting room.
	3. **Staffing**	
	a. Maintain normal staffing operations	
	b. Minimize staff floating on/off unit	
	4. **Serial Patient Testing**	
	a. Immediately isolate and test any symptomatic patient with Strep PCR	
	b. Test all asymptomatic patients on the unit with Strep PCR at day 5-7 based on IPC guidance	Date of planned testing:
	5. **Employee Testing**	
	a. Staff to report symptoms and positive tests to EOHS and stay home if sick.	
	b. Defer to EOHS for recommendations on employee testing	

Supplemental Figure 1: Group A Streptococcal PHARYNGITIS/TONSILITIS Exposure Standard Workflow and Mitigation Plan
Department of Epidemiology and Infection Prevention *

	6. **Miscellaneous**	
	a. ESD will clean high touch surfaces twice a day	
	b. Employees and patients are encouraged to perform frequent hand hygiene	
	c. Employees and patients encouraged to practice respiratory hygiene	
	d. Hallways/groups will program separately (if applicable)	
	e. HEPA filters in positive GAS patient rooms on high setting	
	f. When possible, cohort positive patients in same room or hallway	
	**Duration of interventions:**	**Reassessment date:**

Updates to Initial Mitigation Strategies (for continued cases)

Date/Time	Number of New Positive Patients	Number of New Positive Staff	Total Number of New Cases (Patients + Staff)	Additional Mitigation Strategies

Date/Time	Number of New Positive Patients	Number of New Positive Staff	Total Number of New Cases (Patients + Staff)	Additional Mitigation Strategies
